
JOURNAL INFORMATION
==============================
NLM Title Abbreviation: ScientificWorldJournal
Journal ID: ScientificWorldJournal
NLM Journal ID: 101131163
Journal ID: TSWJ

The Scientific World Journal

ISSN: 2356-6140
EISSN: 1537-744X
Publisher: Wiley

ARTICLE INFORMATION
==============================
PMCID: PMC6009367
PMID: 12806016
DOI: 10.1100/tsw.2002.149
Article ID: 109723
Article version: 1
Subjects: Commentary

Ozone and Plants: Need for a Biologically Based Air Quality Standard

Manning William J.
University of Massachusetts, Amherst, USA
Publication date: 2002
Electronic publication date: 2002 Feb 1
Volume: 2
First page: 282
Last page: 283
Received 2001 Jan 31; Accepted 2001 Jan 31
Copyright: Copyright © 2002 William J. Manning.
Copyright year: 2002
License: This is an open access article distributed under the Creative Commons Attribution License, which permits unrestricted use, distribution, and reproduction in any medium, provided the original work is properly cited.
License URL: https://creativecommons.org/licenses/by/3.0/

Keywords: ozone, plants, air quality, bioindicators, phytotoxicology, AOT40

==============================
Commentary
*TheScientificWorldJOURNAL* (2002) 2, 282–283
ISSN 1537-744X; DOI 10.1100/tsw.2002.149

### Ozone and Plants: Need for a Biologically Based Air Quality Standard

William J. Manning
*University of Massachusetts, Amherst*

*Received January 31; Accepted January 31, 2001; Published February 1, 2002*

**KEY WORDS:** ozone, plants, air quality, bioindicators, phytotoxicology, AOT40

**DOMAINS:** terrestrial environmental toxicology, atmospheric systems, environmental management and policy

For the past 10 years, I have spent parts of late July and early August in central Europe, assessing ozone injury symptom expression on native plants in upland meadows and along forest edges. Much of this work has been done with local colleagues in and near the Tatra Mountains in southern Poland and eastern Slovakia and in the Carpathian Mountains in western Ukraine. Active and passive ozone air monitors and samplers were also used at most of the study sites.

A number of herbaceous annuals, biennials, and perennials and several deciduous shrubs, trees, and vines have been indentified as potential detector (*in situ*) bioindicators for ambient ozone, based on injury symptom expression. Many of these symptoms have been verified on plants grown in our greenhouse ozone exposure chambers. These plants are available as reliable bioindicators in the field.

Monitored concentrations of ambient ozone are used to create maps of the region to locate areas that exceed air quality standards and where native plant injury might occur. Combining cumulative data with GIS techniques results in elegant and accurate depictions of air quality for the region.

Results from our field surveys have varied from year to year, depending on the weather. In cool, wet, cloudy summers, ambient ozone is low and little plant injury occurs. In hot, dry summers, ozone may be high, but available soil moisture is low. Under these conditions, gas exchange is restricted and little ozone is taken up via stomata. Ozone must be taken up by stomata in sufficient concentration to cause injury.

Classic toxicology tells us that exposure of a sensitive organism to a toxicant results in a dose/response function, where exposure equals response. As we have seen, this is not the case for plants growing in the field. Many environmental variables, such as sunlight, air temperature, and soil moisture control stomatal opening and ozone uptake. Concentrations of ozone in the air fluctuate continually. Plants respond to ozone fluxes rather than concentrations of ozone. This results in a dynamic, ever-changing system that has different effects on every plant species.

Given this appreciation of how ambient ozone affects native plants, it is disturbing to realize that there are people in North America and Europe who want to establish air quality standards to protect plants from ozone injury, based only on cumulative hourly ozone concentrations, using a fixed threshold concentration where effects begin. These standards are based on two assumptions:

Email: aamanning@mindspring.com
©2002 with author.

(1) plant exposure to ozone equals plant response, and (2) there is an identifiable uniform threshold value for plant injury caused by ozone.

Cumulative AOT40 standards for ozone are being used and evaluated in Europe. AOT40 is calculated as the sum of the differences between the hourly ozone concentration and the threshold value of 40 ppb (parts per billion v/v) for each hour that the concentration exceeds 40 ppb. AOT40 is cumulative and expressed as ppb hours. Critical cumulative levels of ozone for agricultural crops, forest trees, and natural/semi-natural plant communities have been developed. These critical levels are designated as the cumulative concentrations of ozone above which adverse effects on plants may occur, based on present knowledge. The present knowledge has been derived largely from short-term reductionist and semi-reductionist experiments, which limits extrapolation of results to ambient conditions.

Any threshold cumulative value air quality standard, like AOT40 and SUM 60, for predicting ozone effects on plants that does not include or account for the environmental variables that affect ozone uptake via stomata has no biological basis or relevance. Using critical accumulation levels alone assumes that plant exposure to ozone equals response and can greatly overestimate ozone injury on plants in the field.

Air quality modelers need to begin to include data on environmental variables, such as air temperature, soil moisture, and sunlight in their predictive systems in addition to just monitored ozone data. This can be done locally and efforts should be made to expand an integrated system to regional scales.

Mapping cumulative ozone concentrations is important to locate areas where plant injury is likely to occur. Ground proofing must then be done, using bioindicators to verify and validate plant injury predictions. If this is not done, then air quality standards like AOT40 and SUM 60 have no biological significance and are only exercises in air quality assessment.

**This article should be referenced as follows:**

Manning, W.J. (2002) Ozone and plants: need for a biologically-based air quality standard. *TheScientificWorldJOURNAL* **2**, 282–283.
